# Cardioprotection via vagus nerve stimulation preconditioning: Reducing ischaemia–reperfusion injury and arrhythmic risk

**DOI:** 10.1113/EP092950

**Published:** 2025-11-06

**Authors:** Feng Hu, Yali Wang, Guangyu Li, Guangyu Wang, Qi Zhuang, Jinyao Jiang, Danfeng Hu, Lihui Zheng, Yan Yao, Minhua Zang, Jun Pu

**Affiliations:** ^1^ Department of Cardiology, Renji Hospital, School of Medicine Shanghai Jiaotong University Shanghai China; ^2^ Department of Respiratory Diseases, Renji Hospital, School of Medicine Shanghai Jiaotong University Shanghai China; ^3^ Cardiac Arrhythmia Center, Fuwai Hospital, National Center for Cardiovascular Diseases Chinese Academy of Medical Sciences and Peking Union Medical College Beijing China

**Keywords:** cardiac function, ischaemia–reperfusion injury, myocardial infarction, reperfusion arrhythmias, vagus nerve stimulation

## Abstract

Acute myocardial infarction is a leading cause of morbidity and mortality, with ischaemia–reperfusion (I/R) injury exacerbating myocardial damage. Vagus nerve stimulation (VNS) has been reported to exert cardioprotective effects, but its efficacy in preconditioning against I/R injury requires further investigation. We evaluated the cardioprotective effects of VNS preconditioning in a rat model of acute myocardial infarction with induced I/R injury. Sixty rats were randomized into Pre‐VNS, Control and Sham groups. The Pre‐VNS group received 1 week of low‐level cervical VNS before induction of I/R injury; stimulation was deactivated 30 min before ischaemia. Survival, echocardiographic function, reperfusion arrhythmias, arrhythmia inducibility, infarct size, apoptosis and inflammatory cytokines were assessed. Survival did not differ significantly between Pre‐VNS and Control groups (75.0% vs. 65.0%, *p* = 0.497). However, Pre‐VNS animals exhibited preserved cardiac function, with higher ejection fraction and fractional shortening (*p* < 0.001). VNS preconditioning reduced the incidence of reperfusion arrhythmia during left anterior descending coronary artery ligature release (*p* = 0.006) and decreased the arrhythmia index on programmed stimulation (*p* = 0.003). Infarct size and cardiomyocyte apoptosis were significantly attenuated (*p* < 0.001), accompanied by markedly lower serum interleukin‐1β, interleukin‐6 and tumour necrosis factor‐alpha levels (*p* < 0.001). VNS preconditioning effectively mitigates I/R injury by improving cardiac function, reducing infarct size and arrhythmias, and attenuating inflammatory and apoptotic responses.

## Abstract (highlights)

- **What is the central question of this study?**
  Does vagus nerve stimulation delivered as a preconditioning strategy confer protection against myocardial ischaemia–reperfusion injury?
- **What is the main finding and its importance?**
  Initiation of vagus nerve stimulation 1 week prior to myocardial infarction, with termination at the time of ischaemia, did not improve survival rates post‐infarction. Nevertheless, vagus nerve stimulation preconditioning significantly reduced infarct size and arrhythmia burden, attenuated inflammatory responses and preserved cardiac function even after stimulation was terminated. These findings highlight vagus nerve stimulation preconditioning as a promising prophylactic neuromodulation strategy for mitigating ischaemia–reperfusion injury.

## 1 INTRODUCTION

Acute myocardial infarction (AMI) is a life‐threatening cardiovascular event that remains a leading cause of morbidity and mortality worldwide. Ischaemia–reperfusion (I/R) injury is characterized by the occurrence of arrhythmias, excessive inflammation, calcium overload and microvascular dysfunction. This pathological process is a major contributor to adverse outcomes following AMI and cardiac interventions, making it a crucial target for research aimed at mitigating myocardial damage and improving patient outcomes (Davidson et al., 2019; Toldo et al., 2018; Welt et al., 2024). Both AMI and I/R injury are closely associated with profound autonomic dysfunction, characterized by an exaggerated sympathetic response and diminished parasympathetic activity (Olshansky et al., 2008; Schwartz et al., 1992; Wu and Vaseghi, 2020; Yap and Camm, 1998). This imbalance contributes significantly to the progression from ischaemia to infarction and heightens the risk of arrhythmias and heart failure (Wu and Vaseghi, 2020).

Vagus nerve stimulation (VNS), originally introduced by Dr J. Kiffin Penry in 1988 for treatment of refractory focal epilepsy (Penry and Dean, 1990), has expanded into multiple therapeutic domains, including depression, chronic pain and certain cardiovascular disorders (Austelle et al., 2022; Liu et al., 2020; Shao et al., 2023; Toffa et al., 2020). In the cardiovascular field, VNS has shown promising effects, such as reducing myocardial remodelling, decreasing infarct size and improving post‐infarction cardiac function (Beaumont et al., 2015; Li et al., 2004; Owens et al., 2024; Zhao et al., 2021). Notably, many previous studies used VNS during the occlusion and/or reperfusion stages of an AMI model (Chen et al., 2022; Kiss et al., 2017; Nuntaphum et al., 2018). Because the acute phase of AMI is often accompanied by hypotension and cardiogenic shock, application of VNS at this time might exacerbate haemodynamic instability (Guevara et al., 2024). To address these concerns, the present study was designed to evaluate whether VNS administered 1 week before I/R injury could provide cardioprotective benefits comparable to or greater than those observed with conventional intra‐ischaemic VNS protocols. Specifically, we aimed to evaluate the impact of VNS preconditioning on survival, infarct size, cardiac function and arrhythmia incidence in a rat AMI model.

## 2 MATERIALS AND METHODS

### 2.1 Ethical approval

All procedures were approved by the Animal Ethics Committee of Shanghai Jiao Tong University (approval no. 2024014) and conducted in accordance with the National Institutes of Health *Guidelines for the Care and Use of Laboratory Animals* (8th edition, 2011) and ARRIVE 2.0 guidelines. Animal welfare was carefully safeguarded, and at the end of the experiments the animals were humanely killed by excision of the heart under deep anaesthesia, in accordance with the American Veterinary Medical Association recommendations and institutional protocols.

### 2.2 Animals and experimental groups

Sixty male Sprague–Dawley rats (220–250 g) were obtained from the Experimental Animal Center of Renji Hospital. The animals were housed in specific pathogen‐free conditions with a 12 h–12 h light–dark cycle, ambient humidity maintained at 30%–70% and a stable temperature of 23°C ± 3°C. Standard chow and water were provided ad libitum. Animals were allowed a 7 day acclimation period before experimentation. General anaesthesia was induced with intraperitoneal pentobarbital sodium (50 mg/kg). During surgical procedures, animals were placed on a heating platform to maintain body temperature, and the depth of anaesthesia was verified by the absence of corneal and limb withdrawal reflexes. At the end of the experiments, all animals were humanely killed under deep general anaesthesia, in accordance with institutional guidelines.

All animals were randomly allocated into three groups with distinct interventions. Rats in the Pre‐VNS group (*n* = 20) underwent implantation of a programmable vagus nerve stimulation device, followed by 1 week of active VNS stimulation. After completion of this preconditioning period, the animals were subjected to I/R injury by transient ligation of the left anterior descending coronary artery (LAD). Rats in the Control group (*n* = 20) underwent device implantation identical to the Pre‐VNS group but without activation of stimulation. After the same 1 week interval, they were exposed to LAD ligation and reperfusion to induce I/R injury. Rats in the Sham group (*n* = 20) underwent device implantation and thoracotomy procedures similar to the other groups; however, LAD ligation was not performed, and VNS stimulation was not applied.

### 2.3 Implantation of VNS device

**FIGURE 1:**
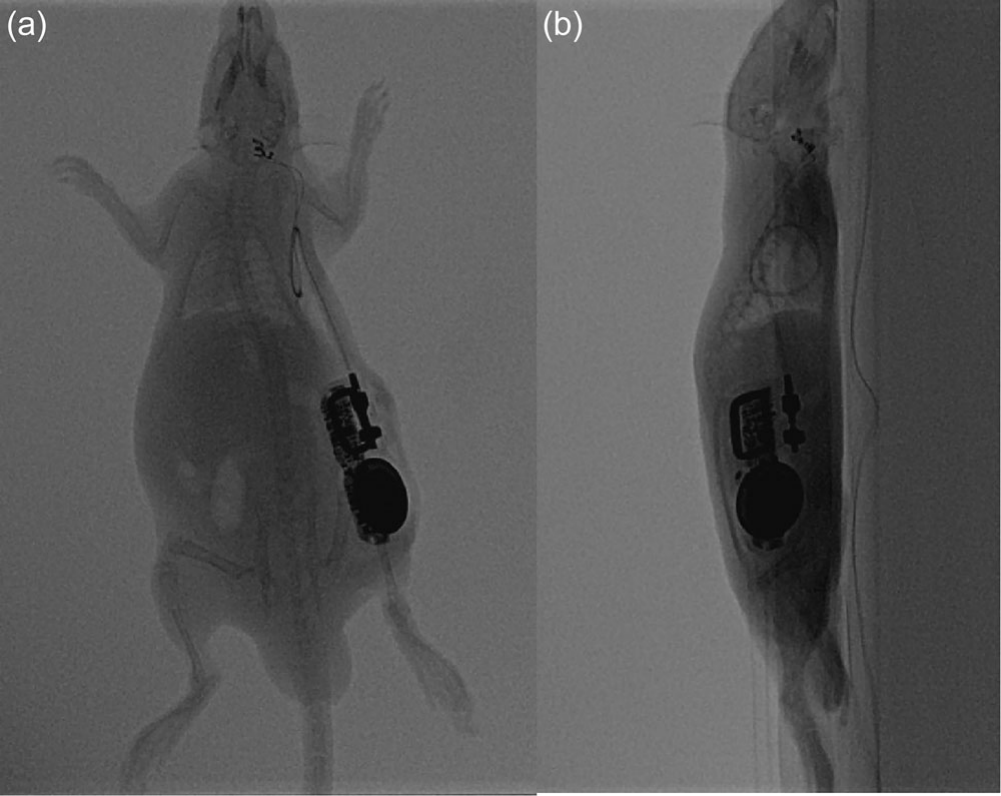
X‐Ray images showing the placement of a vagus nerve stimulator. The pulse generator is implanted subcutaneously in the dorsal abdominal region, and the stimulation electrode, featuring platinum spiral wires, is connected to the right vagus nerve in the cervical region via a subcutaneous tunnel. (a) Anteroposterior view. (b) Lateral view.

**FIGURE 2:**
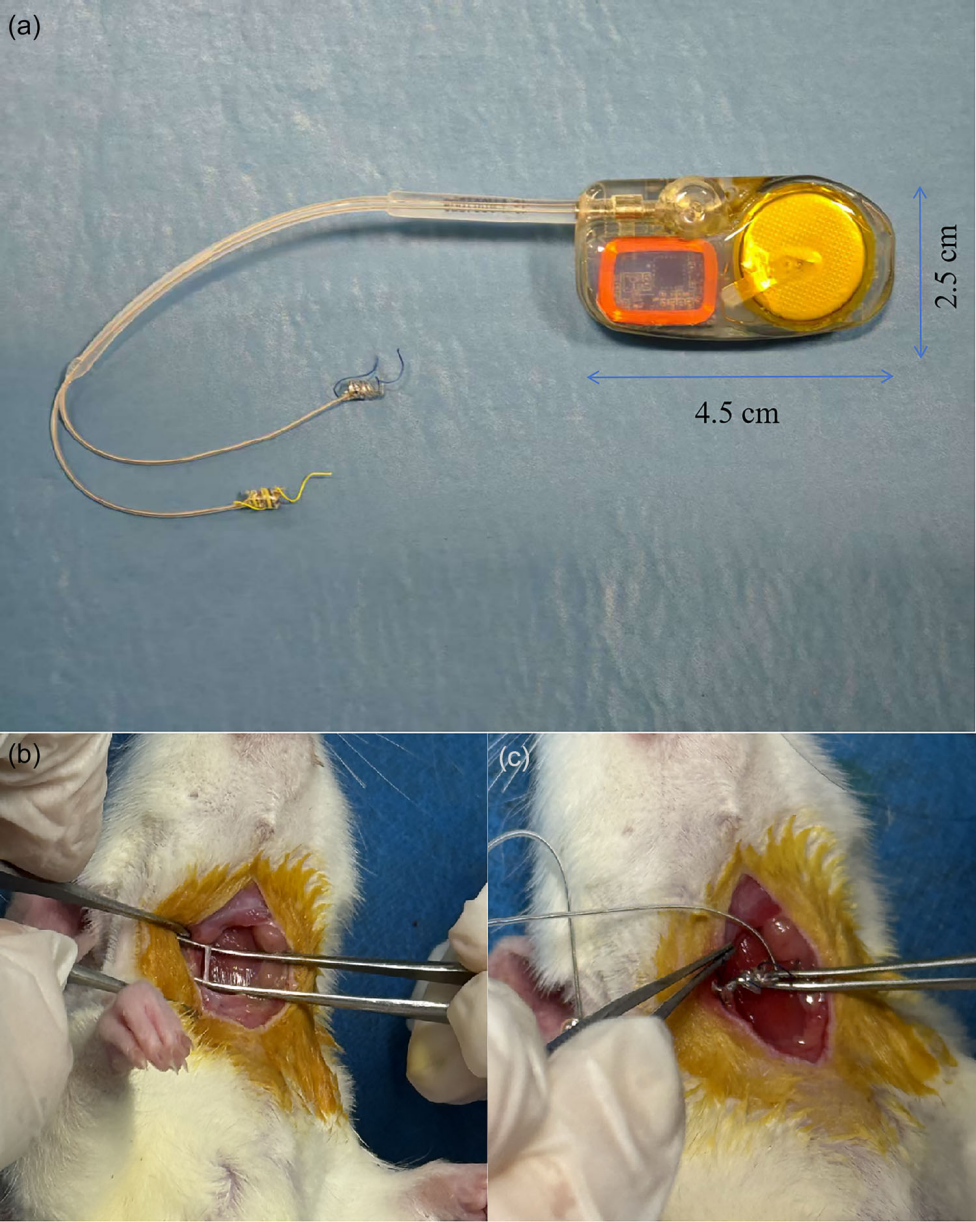
Implantation of the vagus nerve stimulation device in rats. (a) The vagus nerve stimulator pulse generator and stimulation lead. (b) Separation of the cervical vagus nerve. (c) Placement of the electrode cuff around the cervical vagus nerve.

An implantable VNS device (201S, Rishena Co., Ltd, China) consisting of a pulse generator and stimulation electrode with programmable external controls was implanted in the Pre‐VNS and Control groups 1 week before inducing the I/R injury model. Anaesthesia was induced via intraperitoneal pentobarbital injection (50 mg/kg). The pulse generator was implanted subcutaneously in the dorsal abdominal region, and a platinum spiral wire cuff electrode (inner diameter 0.5–0.6 mm, length 3 mm) was tunnelled subcutaneously to the neck and connected to the right vagus nerve (Figures 1 and 2). In the Pre‐VNS group, stimulation parameters were set to 0.2 mA, 0.2 ms pulse width and 20 Hz, delivered in 10 s intervals every minute to achieve a 5%–20% reduction in baseline heart rate. The chosen parameters (0.2 mA, 0.2 ms, 20 Hz and 10 s on–50 s off) were based on prior rodent studies (Hu et al., 2025). In the Control group, the VNS device remained inactive throughout the study. All rats received postoperative analgesia with buprenorphine (0.05 mg/kg, subcutaneously).

### 2.4 Myocardial I/R injury model

Both the Pre‐VNS and Control groups underwent induction of acute myocardial I/R injury. To eliminate potential interference of VNS on arrhythmia development, the VNS device was deactivated 30 min before initiating the I/R injury model. General anaesthesia was induced via intraperitoneal pentobarbital injection (50 mg/kg), followed by endotracheal intubation and mechanical ventilation. During endotracheal intubation and mechanical ventilation, animals were ventilated with room air supplemented with oxygen (approximately 40% O_2_). AMI was induced by transient occlusion of the LAD for 30 min, confirmed by ECG changes, myocardial pallor and localized wall motion abnormalities. The LAD ligature was subsequently released, and the thoracic cavity was closed. Post‐surgery, the animals were placed on a 37°C heated recovery platform. Continuous ECG monitoring was performed using subcutaneous needle electrodes connected to a PowerLab/8SP system (AD Instruments, Australia) during AMI model preparation and for 60 min after reperfusion. From 1 h post‐model establishment throughout the subsequent 24 h, ECG signals were continuously recorded using a wireless ECG telemetry system (Kexin Medical Biotechnology Co., Ltd, Shanghai, China) to document arrhythmic events. In the Sham group, the same surgical procedure was followed, but LAD ligation was omitted.

### 2.5 Echocardiographic measurement

Transthoracic echocardiography was performed before induction of I/R injury and 24 h after reperfusion to evaluate cardiac function. The echocardiographic data were acquired using a Vevo2100 ultrasound platform (Fujifilm VisualSonics Inc., Ontario, Canada), and the measurements were completed by an experienced sonographer blinded to the experimental groups. Key parameters measured included left ventricular end‐systolic diameter, left ventricular end‐diastolic diameter, ejection fraction and fractional shortening.

### 2.6 Ventricular arrhythmia inducibility

Ventricular inducibility testing was performed immediately after echocardiographic measurements during the same experimental session. Programmed electrical stimulation (PES) was conducted using a MicroPace EPS320 stimulator (Micropace EP, Homebush West, NSW, Australia). Under re‐anaesthesia and mechanical ventilation as described in Section 2.4, thoracotomy was repeated for epicardial stimulation. PES was delivered via electrodes secured to the epicardial surface of the right ventricular outflow tract using a steerable electrophysiology catheter (IBI‐81102, Abbott Medical, USA). The protocol consisted of an eight‐beat drive train at a cycle length of 120 ms (S1), followed by up to three extrastimuli (S2–S4) introduced in 5 ms decrements until refractoriness. Stimulation output was set at twice the diastolic threshold. The pacing end point was induction of ventricular tachyarrhythmias. Ventricular tachycardia was defined as at least three consecutive premature ventricular contractions, and ventricular fibrillation was defined as rapid, disorganized ventricular activity. Arrhythmias lasting <30 s were classified as non‐sustained, and those lasting ≥30 s were classified as sustained. Arrhythmia inducibility was graded on an eight‐point scale: 0, non‐inducible; 1, non‐sustained arrhythmia with three extrastimuli; 2, sustained arrhythmia with three extrastimuli; 3, non‐sustained arrhythmia with two extrastimuli; 4, sustained arrhythmia with two extrastimuli; 5, non‐sustained arrhythmia with one extrastimulus; 6, sustained arrhythmia with one extrastimulus; 7, arrhythmia induced during the basic drive; and 8, cardiac arrest prior to pacing. If multiple arrhythmias occurred, the highest score was recorded (Kang et al., 2009).

Upon completion of the PES, blood samples were collected from the abdominal aorta, and the heart was excised under deep anaesthesia, thereby resulting in death of the animals, in accordance with the approved animal care protocols of the Animal Ethics Committee of Shanghai Jiao Tong University.

### 2.7 Biochemical analysis of plasma

Plasma levels of cardiac biomarkers, including cardiac troponin T (cTnT), creatine kinase‐MB (CK‐MB) and lactate dehydrogenase (LDH), were quantified. Inflammatory markers, such as interleukin‐1β (IL‐1β), interleukin‐6 (IL‐6) and tumour necrosis factor‐alpha (TNF‐α), were measured using ELISA kits according to the manufacturer's instructions. The ELISA kits used included cTnT (CSB‐E13646B, Cusabio Technology LLC), CK‐MB (YY‐ELISA0858, Shanghai Yiyan), LDH (C0016, Beyotime, Beijing, China), IL‐1β (PI303, Beyotime, Beijing, China), IL‐6 (PI328, Beyotime, Beijing, China) and TNF‐α (PT516, Beyotime, Beijing, China).

### 2.8 Triphenyltetrazolium chloride (TTC) staining and Haematoxylin and Eosin staining

Myocardial tissues were harvested, frozen at −20°C for 20 min, then sectioned into 1‐mm‐thick slices. The sections were incubated in 2% 2,3,5‐triphenyltetrazolium chloride (TTC) solution at 37°C for 20 min in the dark and subsequently fixed in 4% paraformaldehyde for 24 h. Infarct size was calculated as a percentage of total myocardial tissue using Image Pro Plus v.6.0 software: Infarct size (%) = (infarct area/total heart area) × 100%. For histological analysis, myocardial samples were fixed, paraffin embedded, sectioned at 4 µm thickness, and stained with Haematoxylin and Eosin for examination under a light microscope, focusing on the scar‐border zone of the left ventricle adjacent to the LAD.

### 2.9 Measurements of apoptosis

Myocardial apoptosis was assessed using terminal deoxynucleotidyl transferase dUTP nick‐end labelling (TUNEL) staining. Cardiac tissues were fixed in 4% paraformaldehyde at 4°C for 24 h, dehydrated and paraffin embedded. Sections were deparaffinized, permeabilized, and stained using a TUNEL kit (C1088, Beyotime, Shanghai, China) according to the manufacturer's protocol. Nuclear counterstaining was performed with 4′,6‐diamidino‐2‐phenylindole (DAPI). Images were acquired by a fluorescence microscope (Leica, Wetzlar, Germany), and the percentage of TUNEL‐positive cells within the scar‐border zone of the left ventricular myocardium was quantified in five randomly selected fields.

### 2.10 Statistical analysis

Data were expressed as the mean ± SD. Group comparisons were performed using one‐way ANOVA or Student's *t*‐test for continuous variables. Categorical data were analysed with the χ^2^ test, and survival outcomes were assessed using the log‐rank test. Statistical significance was set at *p* < 0.05.

## 3 RESULTS

### 3.1 Heart rate and survival

In the Pre‐VNS group, heart rate decreased by 7.9% ± 2.6% immediately during stimulation compared with baseline (from 349.2 ± 26.9 to 321.9 ± 31.5 beats/min, *p* < 0.001). One week after initiation of VNS, heart rate remained significantly lower than baseline (324.4 ± 26.0 beats/min, *p* < 0.001). In contrast, the Control group showed no significant difference between baseline and 1 week later (352.6 ± 21.1 vs. 358.7 ± 16.8 beats/min, *p* = 0.305).

**FIGURE 3:**
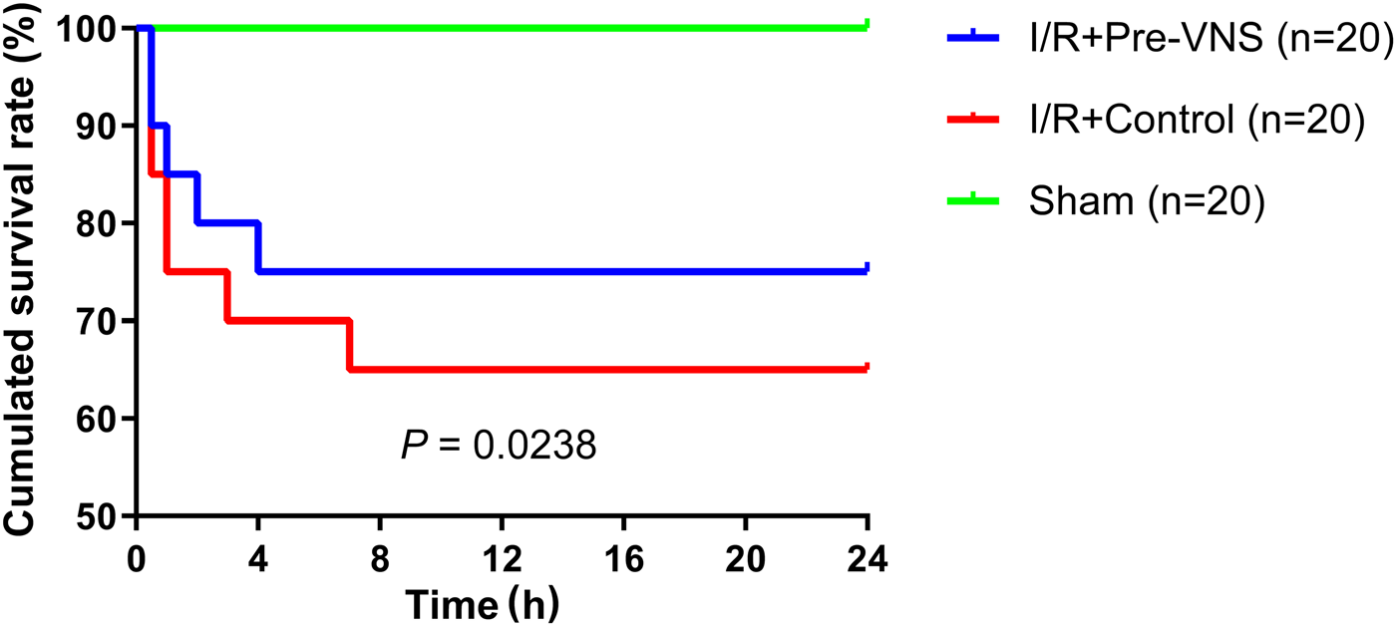
Survival curves for experimental groups. The overall log‐rank test indicated a statistically significant difference amongst the three groups (*p* = 0.024). The survival difference between Pre‐VNS and Control was not statistically significant (75.0% vs. 65.0%, *p* = 0.497). *n* = 20 per group.

As shown in Figure 3, the Sham group maintained 100% (20 of 20) survival throughout the study, whereas the Control group showed the lowest survival rate of 65.0% (13 of 20). The Pre‐VNS group exhibited a survival rate of 75.0% (15 of 20). The overall log‐rank test indicated a statistically significant difference amongst the three groups (*p* = 0.024). Pairwise comparisons revealed that survival was significantly higher in the Sham group compared with both Control (*p* = 0.004) and Pre‐VNS (*p* = 0.018). The difference in survival between Pre‐VNS and Control was not statistically significant (75.0% vs. 65.0%, *p* = 0.497).

### 3.2 Echocardiographic assessment

**FIGURE 4:**
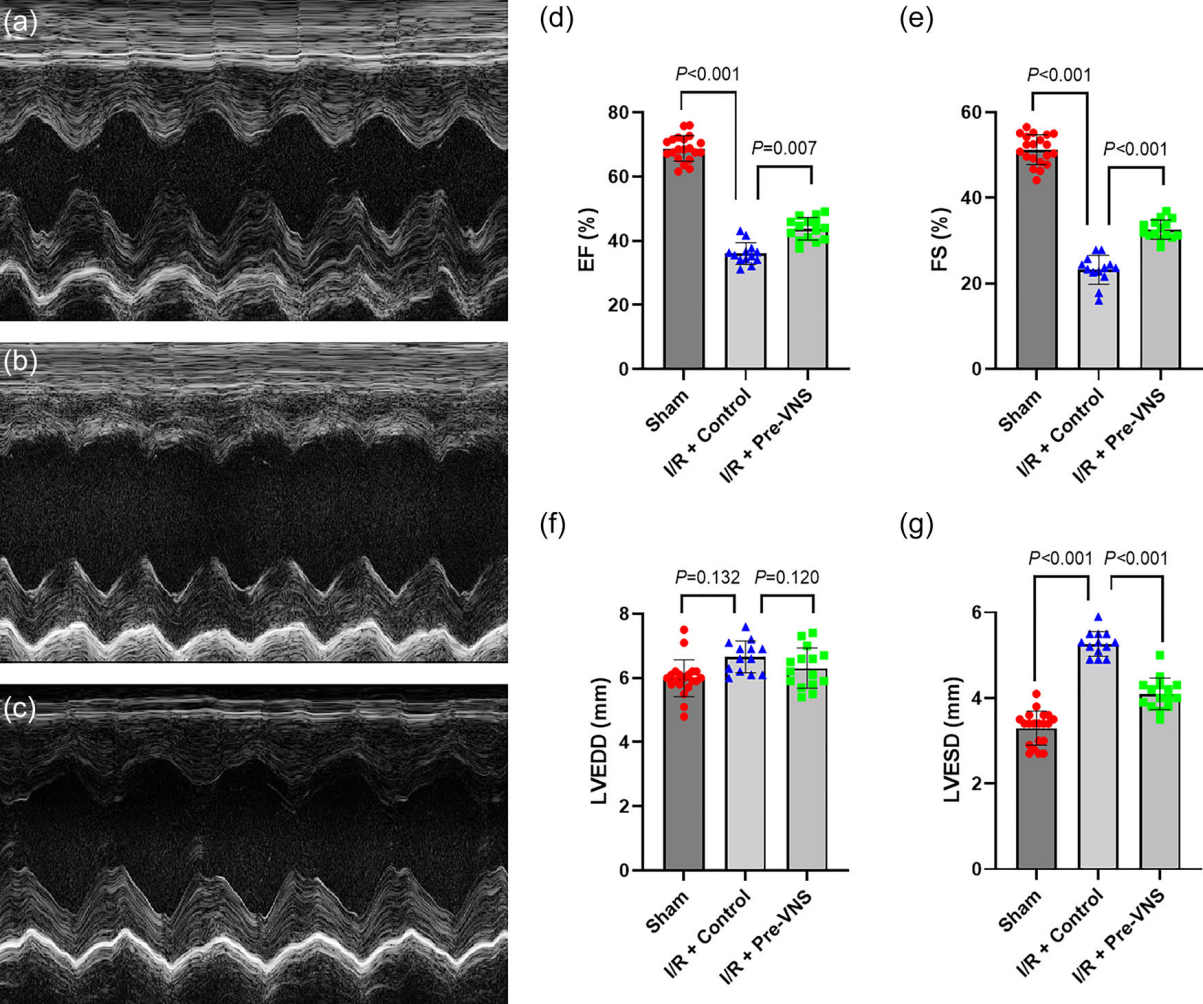
Comparison of echocardiographic and cardiac function parameters. (a–c) Representative M‐mode echocardiographic recordings obtained with two‐dimensional guidance from a short‐axis mid‐ventricular view: (a) Sham group; (b) Control group; and (c) Pre‐VNS group. (d) Comparison of ejection fraction (EF) amongst groups. (e) Comparison of fractional shortening (FS) amongst groups. (f) Comparison of left ventricular end‐diastolic diameter (LVEDD) amongst groups. (g) Comparison of left ventricular end‐systolic diameter (LVESD) amongst groups. *n* = 20 in the Sham group, *n* = 13 in the Control group and *n* = 15 in the Pre‐VNS group.

Echocardiographic analyses are summarized in Figure 4. The Pre‐VNS group demonstrated superior cardiac performance compared with the Control group. Specifically, the ejection fraction was higher in the Pre‐VNS group (45.10% ± 3.81%) compared with the Control group (36.30% ± 3.65%, *p* = 0.007). Fractional shortening was also significantly greater in the Pre‐VNS group (32.70% ± 2.87% vs. 23.40% ± 2.84%, *p* < 0.001). No significant difference in left ventricular end‐diastolic diameter was detected between the Pre‐VNS and Control groups (*p* = 0.132); however, left ventricular end‐systolic diameter was significantly smaller in Pre‐VNS rats (4.05 ± 0.34 vs. 5.17 ± 0.36 mm, *p* < 0.001). Taken together, these findings confirm a protective effect of VNS preconditioning on left ventricular systolic function.

### 3.3 Impact of Pre‐VNS on reperfusion arrhythmias and ventricular arrhythmia inducibility

**FIGURE 5:**
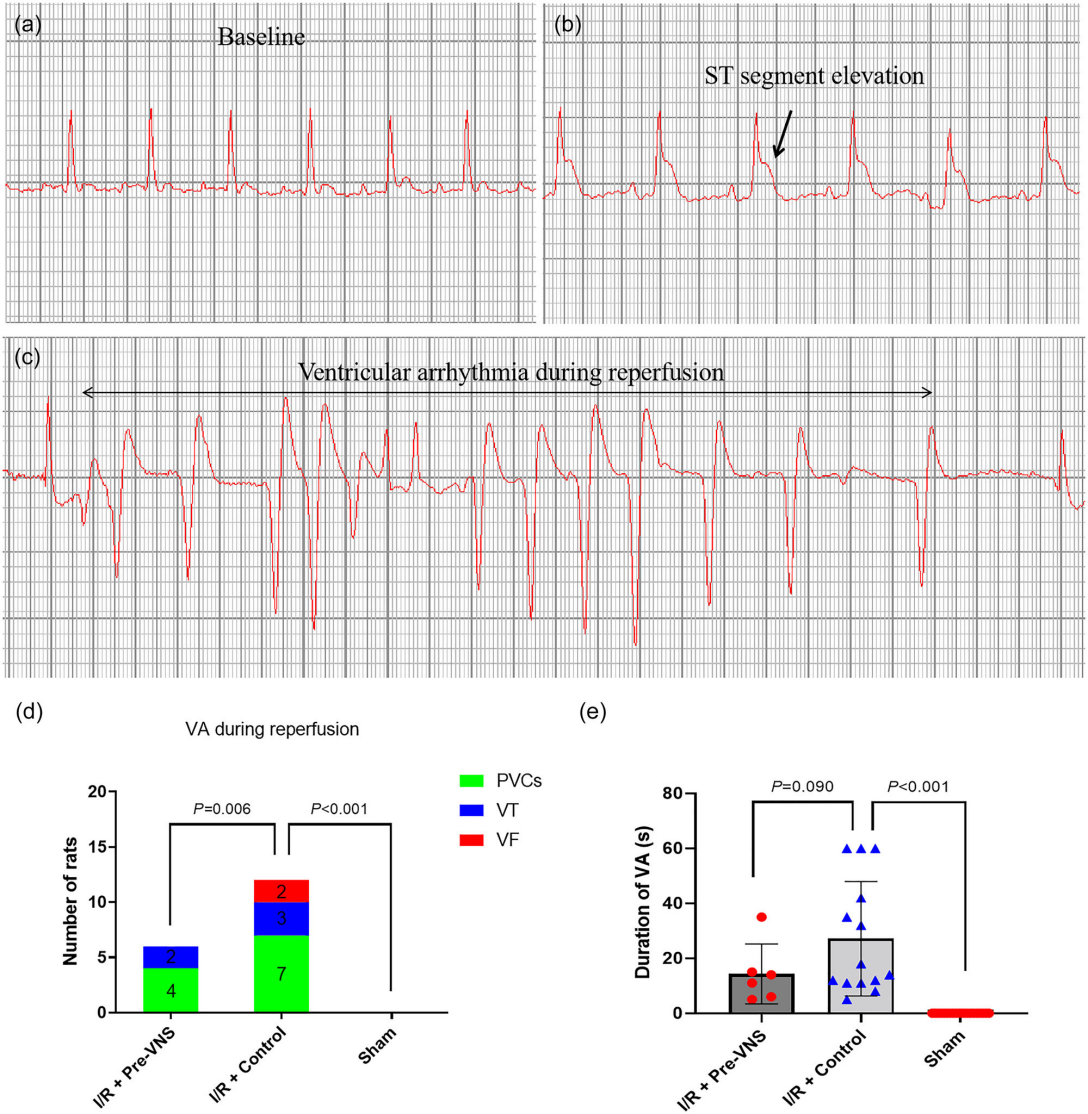
VNS preconditioning inhibits reperfusion arrhythmias. (a) Baseline ECG. (b) ECG showing ST‐segment elevation during LAD ligation. (c) Typical ECG of ventricular arrhythmias during LAD reperfusion. (d) Comparison of the incidence of reperfusion arrhythmias amongst the three groups. (e) Comparison of the duration of reperfusion arrhythmias amongst the three groups. *n* = 20 in the Sham group, *n* = 13 in the Control group, *n* = 15 in the Pre‐VNS group. Abbreviations: I/R, ischaemia–reperfusion; LAD, left anterior descending coronary artery; PVC, premature ventricular contraction; VA, ventricular arrhythmia; VNS, vagus nerve stimulation; VT, ventricular tachycardia.

Following reperfusion, premature ventricular contractions, ventricular tachycardia and ventricular fibrillation were frequently observed in the Control group. As depicted in Figure 5, reperfusion arrhythmias after LAD release were significantly less frequent in the Pre‐VNS group compared with Control animals (*p* = 0.006). The duration of these arrhythmias was not statistically different between the Pre‐VNS group (14.33 ± 10.91 s) and the Control group (27.13 ± 19.66 s, *p* = 0.090).

**FIGURE 6:**
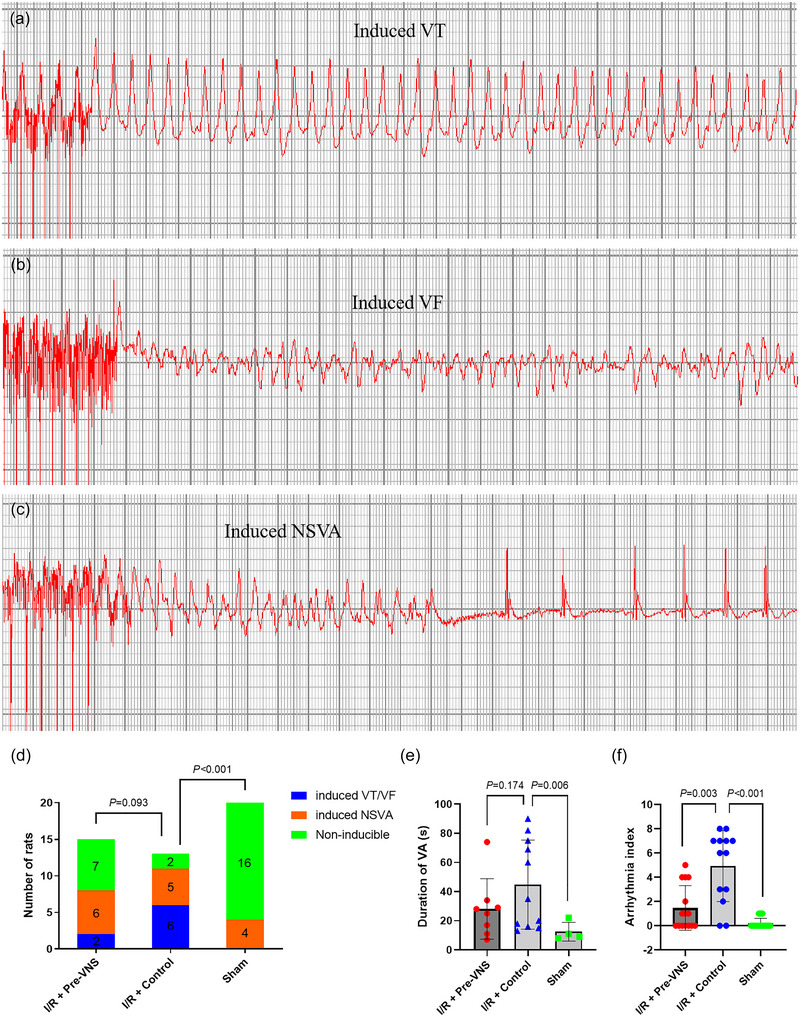
VNS preconditioning reduces ventricular arrhythmia inducibility. (a) Sustained ventricular tachycardia induced during PES. (b) Ventricular fibrillation induced during PES. (c) Non‐sustained ventricular tachycardia induced during PES. (d) Comparison of the incidence of induced VAs amongst the three groups. (e) Comparison of the duration of induced VAs amongst the three groups. (f) Comparison of the arrhythmia index score amongst the three groups. *n* = 20 in the Sham group, *n* = 13 in the Control group and *n* = 15 in the Pre‐VNS group. Abbreviations: I/R, ischaemia–reperfusion; NSVA, non‐sustained ventricular arrhythmias; PES, programmed electrical stimulation; VA, ventricular arrhythmia; VF, ventricular fibrillation; VNS, vagus nerve stimulation; VT, ventricular tachycardia.

Analysis of ventricular arrhythmia inducibility is shown in Figure 6. The prevalence of both sustained and non‐sustained ventricular arrhythmias did not differ significantly between the Pre‐VNS and Control groups (*p* = 0.093). Likewise, the duration of induced ventricular arrhythmias did not differ significantly between the two groups (28.13 ± 20.75 vs. 44.82 ± 30.52 s, *p* = 0.174). However, the arrhythmia index, reflecting the ease of arrhythmia induction, was markedly lower in the Pre‐VNS group compared with the Control group (1.47 ± 1.84 vs. 4.92 ± 2.93, *p* = 0.003).

### 3.4 Effect of Pre‐VNS on myocardial injury and serum cardiac markers

**FIGURE 7:**
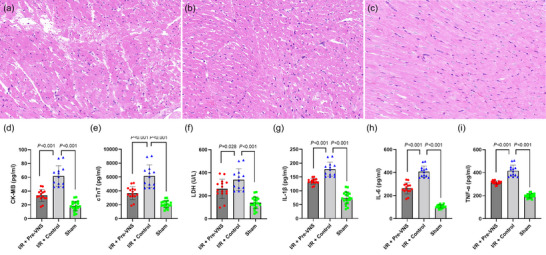
Protective effects of VNS preconditioning on myocardial ischaemia–reperfusion injury: histopathology, serum markers and inflammatory cytokines. (a–c) Representative Haematoxylin‐ and Eosin‐stained myocardial tissue sections: (a) Control group, (b) Pre‐VNS group, (c) Sham group. (d–f) Serum cardiac markers following ischaemia–reperfusion injury: (d) CK‐MB levels were significantly elevated in the Control group compared with the Sham group, but were reduced by VNS preconditioning. (e) cTnT levels were significantly higher in the Control group and mitigated by VNS preconditioning. (f) LDH levels were elevated in the Control group and attenuated in the Pre‐VNS group. (g–i) Serum inflammatory cytokine levels: (g) IL‐1β levels were significantly reduced by VNS preconditioning compared to the Control group; (h) IL‐6 levels were markedly decreased in the Pre‐VNS group; and (i) TNF‐α levels were significantly lowered by VNS preconditioning relative to the Control group. *n* = 20 in the Sham group, *n* = 13 in the Control group and *n* = 15 in the Pre‐VNS group. Abbreviations: CK‐MB, creatine kinase‐MB; cTnT, cardiac troponin T; I/R, ischaemia–reperfusion; IL‐1β, interleukin‐1β; IL‐6, interleukin‐6; LDH, lactate dehydrogenase; TNF‐α, tumour necrosis factor‐alpha; VNS, vagus nerve stimulation.

**FIGURE 8:**
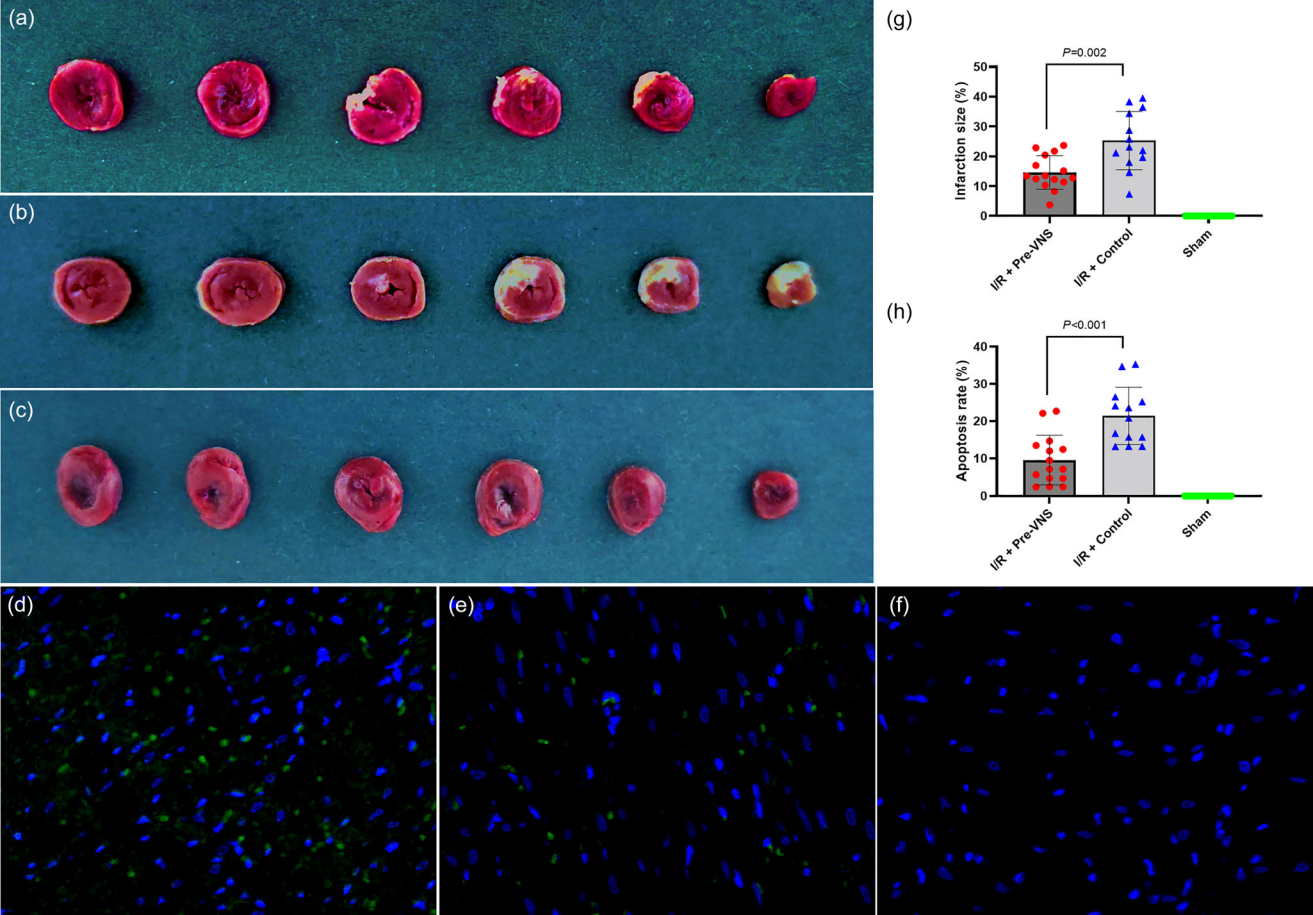
VNS preconditioning reduces infarct size and cardiomyocyte apoptosis. (a–c) Representative images of TTC‐stained heart sections 24 h after reperfusion: (a) Pre‐VNS group; (b) Control group; and (c) Sham group. (d–f) Representative images of TUNEL‐stained heart sections 24 h after reperfusion: (d) Control group; (e) Pre‐VNS group; and (f) Sham group. (g) Quantitative analysis of infarct size ratio. (h) Quantitative analysis of TUNEL staining showing positive apoptotic cells in the scar‐border zone of the left ventricular myocardium. *n* = 20 in the Sham group, *n* = 13 in the Control group and *n* = 15 in the Pre‐VNS group. Abbreviations: I/R, ischaemia–reperfusion; TTC, triphenyltetrazolium chloride; TUNEL, terminal deoxynucleotidyl transferase dUTP nick‐end labelling; VNS, vagus nerve stimulation

As depicted in Figures 7 and 8, I/R injury in the Control group resulted in enlarged infarct size and elevated serum levels of CK‐MB, cTnT and LDH compared with the Sham group (all *p* < 0.05). In contrast, the Pre‐VNS group showed significantly smaller infarct size (14.60% ± 5.64% vs. 25.30% ± 9.78%, *p* = 0.002) and decreased CK‐MB (33.78 ± 8.54 vs. 61.89 ± 14.87, pg/ml, *p* < 0.001), cTnT (3671.10 ± 926.93 vs. 6130.90 ± 1613.87, pg/ml, *p* < 0.001) and LDH (260.40 ± 84.01 vs. 339.80 ± 94.10, U/L, *p* = 0.028) relative to the Control group. Histopathological analysis (Haematoxylin and Eosin staining) corroborated these biochemical findings, revealing that VNS preconditioning preserved myocardial structure and minimized inflammatory cell infiltration.

### 3.5 Effect of Pre‐VNS on serum inflammatory cytokines

The inflammatory mediators IL‐1β, IL‐6 and TNF‐α were markedly elevated in the Control group after I/R injury. However, VNS preconditioning significantly attenuated these increases (Figure 7g–i). Specifically, IL‐1β decreased from 178.50 ± 10.35 to 134.30 ± 10.34 pg/mL (*p* < 0.001), IL‐6 dropped from 408.50 ± 49.01 to 263.30 ± 47.91 pg/mL (*p* < 0.001), and TNF‐α was reduced from 416.10 ± 46.56 to 313.27 ± 15.19 pg/mL (*p* < 0.001). These results underscore the anti‐inflammatory effect exerted by VNS preconditioning in the setting of I/R injury.

### 3.6 Effect of pre‐VNS on cardiomyocyte apoptosis

As illustrated in Figure 8, TUNEL staining revealed a cardiomyocyte apoptosis rate of 21.40% ± 7.69% in the Control group, whereas the Pre‐VNS group exhibited a significantly lower rate of 9.60% ± 6.70% (*p* < 0.001). This reduction in apoptosis aligns with the diminished infarct size and improved functional outcomes noted in the Pre‐VNS group.

## 4 DISCUSSION

In this study, we demonstrated that VNS preconditioning significantly reduced myocardial infarct size, preserved left ventricular function, alleviated reperfusion arrhythmias and mitigated inflammatory responses in a rat I/R model. These results suggest that a preconditioning approach to vagal stimulation might offer substantial cardioprotective effects, extending beyond those observed when VNS is initiated during coronary occlusion or reperfusion (Chen et al., 2022; Kiss et al., 2017; Nuntaphum et al., 2018).

Unlike most previous studies, which focused on VNS applied during coronary occlusion or immediately after reperfusion, we adopted a protocol in which low‐intensity VNS was delivered continuously for 1 week, then deliberately deactivated prior to ischaemia. This strategy was designed to mitigate potential clinical concerns, such as hypotension or cardiac decompensation, which are common in the acute phase of myocardial infarction and could be exacerbated by VNS. By shifting stimulation to a preconditioning phase, the myocardium might benefit from sustained autonomic modulation, enhanced vagal tone and suppression of excessive sympathetic activity; factors that collectively reduce arrhythmogenesis and myocardial injury at the onset of ischaemia (Booth et al., 2024; Cai et al., 2024; Clifford et al., 2024; Guevara et al., 2024).

In clinical practice, however, AMI usually occurs suddenly and unpredictably, making it impractical to perform invasive VNS for 1 week in advance. Nevertheless, with the development of non‐invasive technologies such as transcutaneous auricular VNS and percutaneous VNS (Huffman et al., 2019; Wang, J et al., 2025, 2025), pre‐emptive neuromodulation might become feasible in high‐risk populations identified through predictive algorithms and intelligent monitoring. Prophylactic VNS in such patients could represent a new avenue for early cardioprotection. As a proof‐of‐concept basic study, our work provides mechanistic insights and serves as a reference for future investigations into clinically applicable, non‐invasive VNS strategies for patients at risk of AMI. If translated into practice, a pre‐emptive VNS protocol could be particularly valuable in high‐risk patients undergoing elective Percutaneous coronary intervention (PCI) or cardiac surgery, where I/R injury is anticipated. By modulating autonomic balance before ischaemia, the myocardium might be ‘preconditioned’ to tolerate reperfusion injury better. Although further large‐animal and clinical studies are required, this approach holds promise for reducing lethal arrhythmias, preserving cardiac function and improving both short‐ and long‐term outcomes following revascularization.

VNS exerts its therapeutic effects by modulating the autonomic nervous system, primarily by enhancing parasympathetic (vagal) tone and inhibiting excessive sympathetic activity (Austelle et al., 2024; Booth et al., 2024; Cai et al., 2024; Clifford et al., 2024; Duff et al., 2024; Guevara et al., 2024). This autonomic modulation is particularly crucial during myocardial I/R, where heightened sympathetic activation increases heart rate, blood pressure and myocardial oxygen demand, thereby exacerbating injury (Kiss et al., 2022; Liu et al., 2023; Longhurst et al., 2001). In contrast, vagal activation reduces heart rate, myocardial workload and oxygen consumption, collectively mitigating tissue damage. For instance, Nuntaphum et al. (2018) demonstrated in an I/R animal model that VNS significantly reduced infarct size, decreased the incidence of ventricular fibrillation and attenuated myocardial injury and cardiomyocyte apoptosis. Likewise, Zhao et al. (2013) reported that VNS diminished infarct size, improved cardiac function and alleviated vasomotor dysfunction and endothelial damage in mesenteric arteries during myocardial I/R. Distinct from previous studies that applied VNS during LAD occlusion or immediately after reperfusion, our protocol involved administering VNS continuously for 1 week prior to inducing I/R, with stimulation deactivated before LAD occlusion via external programming. Our findings indicate that this pre‐emptive VNS treatment offers cardioprotection comparable to earlier intra‐ischaemic approaches.

A particularly notable finding was the significant reduction in infarct size in the VNS‐preconditioning group (14.60% ± 5.64%) compared with control animals (25.30% ± 9.78%). This result aligns with earlier studies by Chen et al. (2016), Kiss et al. (2017) and Nuntaphum et al. (2018), which demonstrated the infarct‐limiting effects of VNS in various I/R models. The observed reduction in infarct size is likely to be attributable to improved myocardial oxygen supply, enhanced microcirculatory function and diminished reperfusion injury, thereby preventing further tissue necrosis and apoptosis. Additionally, TUNEL staining revealed significantly fewer apoptotic cardiomyocytes in the Pre‐VNS group, consistent with findings by Katare et al. (2009), suggesting that VNS inhibits caspase‐3 activation and reduces ischaemia‐induced myocardial apoptosis.

Ventricular arrhythmias (particularly sustained ventricular tachycardia and ventricular fibrillation) are major complications following AMI and reperfusion that contribute to elevated morbidity and mortality (Anter, 2012; Bigger et al., 1984; Gottlieb et al., 1988; Juul‐Möller et al., 1988; Shi et al., 2022; Zhao et al., 2022). Previous studies have demonstrated anti‐arrhythmic effects of VNS when applied during LAD occlusion and reperfusion (Arimura et al., 2017; Hadaya et al., 2023; Lai et al., 2019; Nederhoff et al., 2019; Yu et al., 2017; Zhao et al., 2021), whereas our study found that VNS preconditioning significantly reduced the incidence of reperfusion arrhythmias (30% in the VNS group vs. 70% in control animals) and shortened their duration, as reflected by a lower arrhythmia index. This anti‐arrhythmic benefit is likely to arise from the capacity of VNS to restore autonomic balance, counteracting pro‐arrhythmic sympathetic overactivity and stabilizing myocardial electrical activity.

The inflammatory response plays a pivotal role in myocardial injury following I/R, with rapid release of pro‐inflammatory cytokines (such as IL‐1β, IL‐6 and TNF‐α) leading to leucocyte infiltration, endothelial dysfunction and oxidative stress (Francisco and Del Re, 2023; Wu et al., 2018). Prior studies have shown that vagal activation can reduce cytokine release and subsequent tissue damage by modulating the inflammatory response (Brock et al., 2021; Calvillo et al., 2011; Zhang et al., 2016). In agreement with these observations, our study demonstrated that VNS preconditioning significantly lowered plasma levels of IL‐1β, IL‐6 and TNF‐α, thereby reinforcing the anti‐inflammatory effects of VNS observed in other organ systems.

Although we did not directly measure autonomic indices or molecular changes during the preconditioning phase, several plausible mechanisms might explain why cardioprotection persisted after cessation of stimulation. Sustained vagal modulation is supported by the observation that heart rate remained significantly lower 1 week after VNS initiation, and despite deactivation of the device prior to I/R, arrhythmia suppression and infarct limitation were still observed. Literature indicates that transient vagal activation can engage muscarinic and α7 nAChR‐mediated pathways, reduce pro‐inflammatory cytokines, stabilize mitochondria and blunt oxidative stress (Xiong et al., 2010; Zhao et al., 2013). In addition, emerging evidence highlights that VNS might suppress TLR4/NF‐κB signalling, inhibit ferroptosis and pyroptosis, and activate protective metabolic cascades (Xu et al., 2023), all of which might persist beyond the stimulation window. These mechanisms provide a biologically plausible basis for the lasting benefits of VNS preconditioning and warrant future investigation.

Several limitations must be acknowledged. First, we used a rat model; thus, extrapolation to human AMI should be approached with caution. Second, our study focused on 24 h outcomes, and the effects of VNS preconditioning on long‐term remodelling or survival remain to be addressed. Third, although we established a single set of stimulation parameters based on previous research, the optimal ‘dose’ (intensity, frequency and duration) for clinical translation warrants further investigation. Fourth, initiating cervical VNS 1 week before MI is not directly applicable to clinical practice; however, as a proof of concept it suggests potential prophylactic use of non‐invasive VNS in high‐risk patients, such as those undergoing PCI or cardiac surgery, where I/R injury is anticipated. Finally, device implantation and early stimulation itself could alter the baseline physiology of rats, which might not fully replicate human clinical scenarios.

## 5 CONCLUSION

In conclusion, this study demonstrates that VNS preconditioning before ischaemia significantly reduces myocardial infarct size, improves cardiac function and mitigates both reperfusion arrhythmias and inflammatory responses in a rat model of AMI with I/R injury. The concept of preconditioning VNS offers a new therapeutic avenue for managing myocardial ischaemic events, potentially improving acute and long‐term outcomes in patients with AMI.

## AUTHOR CONTRIBUTIONS

Conceptualization: Feng Hu and Jun Pu. Data curation: Feng Hu, Yali Wang, Guangyu Li, Guangyu Wang, Qi Zhuang, Jinyao Jiang and Danfeng Hu. Formal analysis: Yali Wang, Guangyu Li and Qi Zhuang. Investigation: Yali Wang, Guangyu Li, Guangyu Wang, Guangyu Wang, Jinyao Jiang and Danfeng Hu. Visualization: Feng Hu. Methodology: Feng Hu, Lihui Zheng, Minhua Zang and Jun Pu. Writing—original draft: Feng Hu and Yali Wang. Supervision: Lihui Zheng, Yan Yao and Minhua Zang. Writing—review and editing: Lihui Zheng, Yan Yao, Minhua Zang and Jun Pu. Resources: Yan Yao. Project administration, supervision, funding acquisition: Jun Pu. All authors reviewed and approved the final manuscript. Each author accepts responsibility for the integrity of the work as a whole and agrees to address any issues concerning accuracy or reliability should they arise. Every individual listed as an author meets the established criteria for authorship, and no eligible contributors have been omitted.

## CONFLICT OF INTEREST

None declared.

## Data Availability

The data generated or analysed for the study are available from the corresponding author on reasonable request.
